# Prognostic implications of aspiration pneumonia in patients with community acquired pneumonia: A systematic review with meta-analysis

**DOI:** 10.1038/srep38097

**Published:** 2016-12-07

**Authors:** Kosaku Komiya, Bruce K. Rubin, Jun-ichi Kadota, Hiroshi Mukae, Tomohiro Akaba, Hiroshi Moro, Nobumasa Aoki, Hiroki Tsukada, Shingo Noguchi, Nobuaki Shime, Osamu Takahashi, Shigeru Kohno

**Affiliations:** 1 Department of Pediatrics, Virginia Commonwealth University School of Medicine, 1217 East Marshall Street: KMSB, Room 215 Richmond, Virginia 23298, USA; 2 Respiratory Medicine and Infectious Diseases, Oita University Faculty of Medicine, 1-1 Idaigaoka, Hasama-machi, Yufu, Oita, 879-5593, Japan; 3 Clinical Research Center of Respiratory Medicine, Tenshindo Hetsugi Hospital, 5956 Nihongi, Nakahetsugi, Oita, 879-7761, Japan; 4 Second Department of Internal Medicine, Nagasaki University School of Medicine, 1-7-1 Sakamoto, Nagasaki, 852-8501, Japan; 5 Department of Respiratory Medicine and Infectious Diseases, Niigata University Graduate School of Medical and Dental Sciences, 757 Asahi-machi, Chuo-ku, Niigata, 951-8510, Japan; 6 Department of Respiratory Medicine/Infectious Disease, Niigata City General Hospital, 463-7 Shumoku, Chuo-ku, Niigata, 950-1197, Japan; 7 Department of Respiratory Medicine, University of Occupational and Environmental Health, 1-1 Idaigaoka, Yahatanishi-ku, Kitakyushu, 807-8555, Japan; 8 Department of Emergency and Critical Care Medicine, Institute of Biomedical & Health Sciences, Hiroshima University Advanced Emergency and Critical Care Center, Hiroshima University Hospital, 1-2-3 Kasumi, Minami-ku, Hiroshima, 734-8553, Japan; 9 Center for Clinical Epidemiology, St. Luke’s Life Science Institute, 10-1 Akashicho, Chuo-ku, Tokyo, 104-0044, Japan

## Abstract

Aspiration pneumonia is thought to be associated with a poor outcome in patients with community acquired pneumonia (CAP). However, there has been no systematic review regarding the impact of aspiration pneumonia on the outcomes in patients with CAP. This review was conducted using the MOOSE guidelines: Patients: patients defined CAP. Exposure: aspiration pneumonia defined as pneumonia in patients who have aspiration risk. Comparison: confirmed pneumonia in patients who were not considered to be at high risk for oral aspiration. Outcomes: mortality, hospital readmission or recurrent pneumonia. Three investigators independently identified published cohort studies from PubMed, CENTRAL database, and EMBASE. Nineteen studies were included for this systematic review. Aspiration pneumonia increased in-hospital mortality (relative risk, 3.62; 95% CI, 2.65–4.96; P < 0.001, seven studies) and 30-day mortality (3.57; 2.18–5.86; P < 0.001, five studies). In contrast, aspiration pneumonia was associated with decreased ICU mortality (relative risk, 0.40; 95% CI, 0.26–0.60; P < 0.00001, four studies). Although there are insufficient data to perform a meta-analysis on long-term mortality, recurrent pneumonia, and hospital readmission, the few reported studies suggest that aspiration pneumonia is also associated with these poor outcomes. In conclusion, aspiration pneumonia was associated with both higher in-hospital and 30-day mortality in patients with CAP outside ICU settings.

The incidence of pneumonia and pneumonia mortality are both greater in the elderly population^1^,^2^, and as human life expectancy continues to increase, it is anticipated that pneumonia deaths will also increase. The most common type of pneumonia in the elderly is aspiration pneumonia^3^,^4^. Aspiration pneumonia can develop after the inhalation of pathogenic bacteria into the lower respiratory tract, and it can present as ventilator-associated pneumonia, hospital-acquired pneumonia, or as community acquired pneumonia (CAP)^5^. The prevalence rate of aspiration pneumonia is estimated to be 5% to 24% in subjects with CAP^6^,^7^,^8^,^9^.

Aspiration pneumonia can be defined as pneumonia in patients who have aspiration risk. Risk factors for aspiration include impaired consciousness, weakness, swallowing difficulties, esophageal dysfunction or mechanical obstruction, and sedation^4^,^10^,^11^. However, overt aspiration is generally not witnessed^12^,^13^, and aspiration alone cannot fully explain the development of pneumonia^14^. Most healthy subjects passively aspirate oropharyngeal secretions during night, but their cough reflex, mucociliary clearance, and immune system usually prevents the development of pneumonia^15^.

Healthcare-associated pneumonia (HCAP) was defined in the 2005 American Thoracic Society/Infectious Diseases Society of America guidelines to identify patients at increased risk for infection due to multidrug-resistant pathogens in CAP^16^. While broad spectrum antibiotic therapy has not been shown to improve the outcome of patients with HCAP^17^,^18^,^19^, other host factors including age, comorbidities and aspiration risk are associated with higher mortality^17^,^20^,^21^,^22^,^23^. In this systematic review, we selected studies using defined diagnostic criteria for aspiration pneumonia to survey the prevalence of aspiration pneumonia and evaluate the relationship between aspiration pneumonia and mortality, recurrent pneumonia, and repeat hospital admission in subjects with CAP.

## Methods

This systematic review was conducted using the Preferred Reporting Items for Systematic Reviews and Meta-Analyses (PRISMA) and the Meta-analysis of Observational Studies in Epidemiology (MOOSE) guidelines^24^.

### Definitions and inclusion criteria

Aspiration pneumonia is defined as pneumonia in subjects with aspiration risk signified by impaired conscious^25^, neurological disease^26^,^27^, esophageal dysfunction or esophageal mechanical obstruction^28^,^29^, or aspiration witnessed during eating or vomiting^4^,^10^,^11^. We included studies in adults aged 15 years and older with specific outcomes that included mortality, recurrent pneumonia, or hospital readmission and we compared these in subjects with “non-aspiration” pneumonia defined as CAP in subjects without aspiration risk. In all but one of the studies we identified, aspiration pneumonia was studied as a risk factor in *all* subjects with CAP including those with HCAP. We could neither perform a multivariate-analysis on a single study, nor could we compare these results with those that combine HCAP with non-HCAP subjects, so this study was excluded from the analysis.

### Search criteria

We identified published cohort studies written in English from the PubMed database using the search terms: “community acquired pneumonia [All Fields] OR healthcare associated pneumonia [All Fields] AND aspiration [All Fields]”, from the Cochrane Central Register of Controlled Trials (CENTRAL) database using the search terms: “community acquired pneumonia AND aspiration” and “healthcare associated pneumonia AND aspiration”, and from the EMBASE using the search terms: “community acquired pneumonia AND aspiration” or “healthcare associated pneumonia AND aspiration” (accessed on August 31, 2016). Studies published only in abstract form were excluded because the methods and results could not be fully assessed. Full texts of articles were further evaluated by three investigators (KK, TA and JK).

### Data extraction

We extracted the following information from included studies: study design, sample size, inclusion and exclusion criteria, subject demographics, prevalence rate of aspiration pneumonia, type of outcome, type of statistical analysis and other significant predictive factors for each outcome.

### Assessing risk of bias

The risk of bias in the included studies was assessed according to the recommendations outlined in the Cochrane Handbook for Systematic Reviews of Interventions Version 5.1.0. and MOOSE guidelines for the following items: selection, performance, detection, attrition, and publication bias^24^. Each study included in this review was assessed for quality as good, moderate, or poor based on biases using the modified Hayden’s criteria^30^. Disagreements among the investigators were resolved by review of the assessments to reach consensus.

### Data analysis

We conducted meta-analysis by the outcomes of studies as follows: in-hospital mortality, and 30-day mortality. ICU mortality, long-term mortality, readmission and recurrence, and treatment failure. Outcomes were pooled using Mantel-Haenszel risk ratios, and the precision of the estimates was expressed as the 95% confidence interval (CI). Statistical heterogeneity was assessed using the Higgins *I*^2^ tests. A random-effects model was used when significant heterogeneity was found. Publication bias was assessed by examination of funnel plots^31^. Statistical significance was defined by a P value < 0.05 for all analyses. The meta-analysis was performed with the Review Manager ver. 5.3 software program (The Nordic Cochrane Centre, The Cochrane Collaboration).

## Results

### Database search and characteristics of included studies

**Figure 1:**
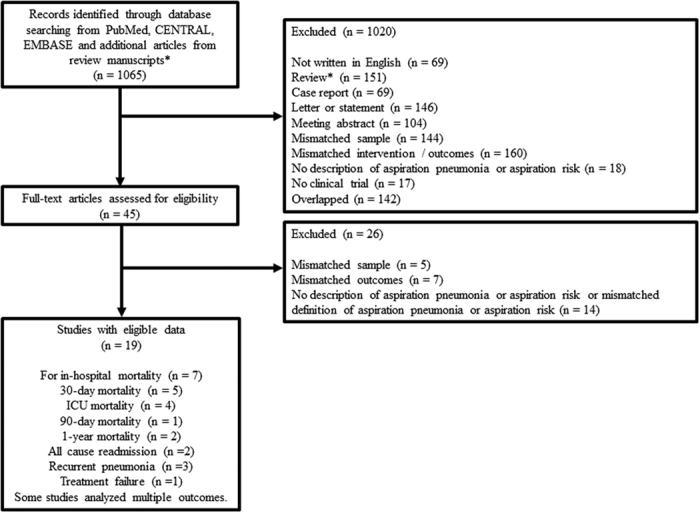
Flow diagram of the study selection.

We identified 1065 studies through PubMed, CENTRAL database, EMBASE, and additional studies from review articles. We then excluded 1020 studies as the abstract did not meet the inclusion criteria. We excluded 26 of the remaining 45 records after retrieving and inspecting the full text. The reasons for exclusion were: the population included inappropriate subjects (n = 5)^32^,^33^,^34^,^35^,^36^, mismatched outcomes (n = 7)^23^,^37^,^38^,^39^,^40^,^41^,^42^, no description of aspiration pneumonia or the definition of aspiration pneumonia did not meet *a priori* criteria (n = 14)^43^,^44^,^45^,^46^,^47^,^48^,^49^,^50^,^51^,^52^,^53^,^54^,^55^,^56^. The process of the study selection is shown in Fig. 1.

We finally included 19 studies in this systematic review: a second analysis of a multicenter retrospective international database of patients with CAP using propensity score analysis^57^, a secondary analysis of a prospective observational study^58^, four prospective observational studies^59^,^60^,^61^,^62^, ten retrospective observational studies^21^,^63^,^64^,^65^,^66^,^67^,^68^,^69^,^70^,^71^, a combined a retrospective derivation cohort and a prospective validation cohort^72^, two other combined a retrospective and a prospective observational studies^73^,^74^. These studies were published from the USA (n = 5), France (n = 4), Japan (n = 4), Spain (n = 3), United Kingdom (n = 1), Canada (n = 1) and Switzerland (n = 1). They were assessed quality based on the modified Hayden’s criteria: good (n = 3)^57^,^58^,^72^, moderate (n = 11)^21^,^61^,^62^,^63^,^64^,^66^,^67^,^69^,^71^,^73^,^74^ or poor (n = 5)^59^,^60^,^65^,^68^,^70^. The percentage of subjects with aspiration pneumonia ranged from 1.5 to 50.4%. All studies diagnosed aspiration pneumonia on clinical grounds, and 9 studies also required radiographic evidence of involvement of a dependent pulmonary segment^21^,^40^,^60^,^62^,^63^,^67^,^68^,^72^,^73^. Some studies performed video fluoroscopy^58^,^59^,^71^. The wide range reported for the prevalence rate of aspiration pneumonia was, no doubt, influenced by subject selection criteria, how “aspiration pneumonia” was clinically defined, and by the judgment of the clinicians involved in making the diagnosis. Outcomes of these studies were classified as follows: 30-day mortality (n = 5)^21^,^58^,^60^,^65^,^66^, in-hospital mortality outside of the ICU (n = 7)^57^,^59^,^63^,^64^,^69^,^70^,^71^, ICU mortality (n = 4)^67^,^72^,^73^,^74^, 90-day mortality (n = 1)^68^, 1-year mortality (n = 2)^58^,^69^, all cause readmission (n = 2)^58^,^66^, recurrent pneumonia (n = 3)^58^,^61^,^63^, and treatment failure (n = 1)^62^. Three of 19 studies evaluated multiple outcomes^50^,^58^,^63^. Although all studies except for two^57^,^68^ described bacteriological findings, none required the detection of *anaerobic* bacteria for the definition of aspiration pneumonia.

### In-hospital mortality

**Figure 2:**
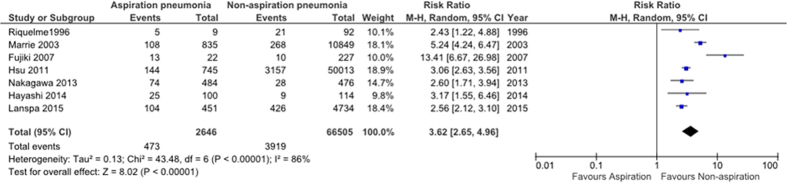
Pooled mean difference for in-hospital mortality with 95% confidence intervals for eligible studies.

Seven studies (69,129 subjects) evaluated in-hospital mortality outside of the ICU^57^,^59^,^63^,^64^,^69^,^70^,^71^. Mortality in patients with aspiration was significantly higher than that in non-aspiration pneumonia (Table 1). Meta-analysis indicated that aspiration risk increased in-hospital mortality (relative risk, 3.62; 95% CI, 2.65–4.96; P < 0.00001; *I*^2^ = 86%) as shown in Fig. 2. Most of the included studies individually assessed the impact of aspiration pneumonia using multivariate analysis (Table 1). Three of these studies found that aspiration pneumonia independently increased in-hospital mortality after multivariate adjustments. Hayashi *et al*. showed that the CURB-65 score; a scoring system for predicting mortality in CAP (HR 1.617; 95% CI 1.236–2.117), and Eastern Cooperative Oncology Group performance status (ECOG PS) (1.476; 1.042–2.090) were associated with higher in-hospital mortality and after adjusting for these, aspiration pneumonia was no longer a risk factor^63^.

### 30-day hospital mortality

**Figure 3:**
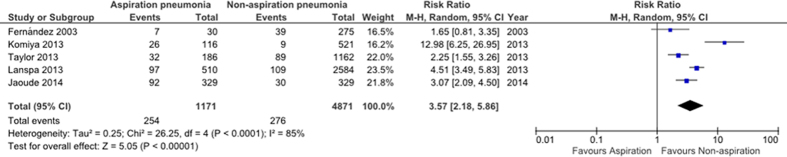
Pooled mean difference for 30-day mortality with 95% confidence intervals for eligible studies.

Five studies (6,042 subjects) analyzed 30-day mortality^21^,^58^,^60^,^65^,^66^. The mortality in patients with aspiration risk was significantly higher than that in non-aspiration pneumonia in four of five studies (Table 2). Fernandez *et al*. included only patients aged over 80 years^60^. In the meta-analysis including this study, aspiration risk increased in-hospital mortality (relative risk, 3.57; 95% CI, 2.18–5.86; P < 0.00001; *I*^2^ = 85%) as shown in Fig. 3. When the study was excluded, the relative risk increased to 4.15 (95% CI, 2.48–7.01; P < 0.00001; *I*^2^ = 86%). While two studies indicated that aspiration risk significantly increased 30-day mortality^21^,^65^, the other studies did not retain statistical significance after adjustment^58^,^60^,^66^. CURB-65 score (HR 1.495; 95% CI 1.033–2.163)^21^, moderate or severe liver disease (9.21; 3.16–26.86) and leukocytosis (4.47; 2.27–8.82) were additional risk factors for 30-day mortality^65^. In studies that showed that aspiration pneumonia was not associated with 30-day mortality in multivariate analysis^58^,^60^, congestive heart failure (OR 2.05; 95% CI 1.31–3.20), pneumonia severity index (PSI) score (2.64; 2.01–3.45), and ECOG PS (1.57; 1.34–1.84)^58^, shock (10.69; 1.33–86.27), respiratory failure (3.50; 1.03–11.96), renal failure (5.83; 2.32–14.68), and Gram-negative pneumonia (20.27; 1.01–410.59)^60^ were associated with higher 30-day mortality.

### ICU mortality

**Figure 4:**
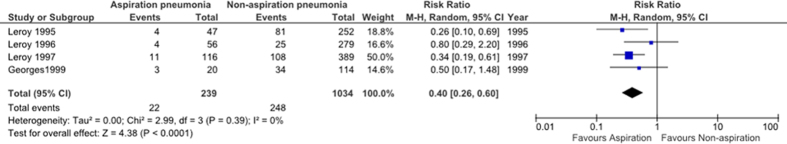
Pooled mean difference for ICU mortality with 95% confidence intervals for eligible studies.

Five studies including 1,644 subjects analyzed ICU mortality^67^,^72^,^73^,^74^. These studies were all published from the same research group in France. Paradoxically, the mortality in subjects with aspiration pneumonia was significantly *lower* than that in those with non-aspiration pneumonia in two of these studies (Table 3), and aspiration pneumonia was associated with decreased ICU mortality (relative risk, 0.40; 95% CI, 0.26–0.60; P < 0.00001) (Fig. 4). One of these studies showed that aspiration risk was a better prognostic factor for ICU survival compared to non-aspiration pneumonia in the multivariate analysis^72^. This study also identified other prognostic factors for ICU mortality including ineffective antimicrobial therapy (prognostic score resulted from validation cohort, +1.5), immunosuppression (+1.38), and higher organ system failure score (OSFS) (+0.64)^75^.

### 90-day mortality and one year mortality

One study evaluated 90-day mortality^68^, and two assessed one year mortality after discharge^58^,^69^. Aspiration pneumonia increased one year mortality (40.4% in aspiration pneumonia *vs* 22.1% in non-aspiration pneumonia, p < 0.001)^69^. The other studies showed that aspiration pneumonia was significantly associated with 90-day mortality (HR 3.09; 95% CI 1.90–5.03)^68^ or one-year mortality (HR 1.73; 95% CI 1.15–2.58)^58^ in each multivariate analysis. Mortensen *et al*.^68^ also found that age (1.64; 1.39–1.93), hypothermia (1.90; 1.03–3.49), liver disease (3.88; 1.18–12.70), white blood cell count <400/uL (2.99; 1.12–8.00), serum urea nitrogen level >30 mg/dL (2.44; 1.62–3.68), and arterial oxygen tension <60 mHg (1.99; 1.32–3.00) were associated with increased 90-day mortality.

### All cause readmission and recurrent pneumonia

We identified no study specifically evaluating on readmission for pneumonia, but 2 studies assessed all cause readmission rate^58^,^66^ and 3 studies analyzed recurrent pneumonia^58^,^61^,^63^ (Table 4). Two studies showed that aspiration risk was associated with increased readmission rate after multivariate analysis, and 2 studies identified that aspiration risk was associated with a higher rate of recurrent pneumonia^58^,^63^. One study showed that age (OR, 2.182; 95% CI 1.370–3.475), lack of pneumococcal vaccination (1.909; 1.302–2.798), COPD (1.534; 1.021–2.303) and corticosteroid therapy >20 mg/day (1.971; 1.047–3.713) but not aspiration risk were associated with recurrent pneumonia^61^.

### Treatment failure

One research group analyzed the association between aspiration risk and treatment failure; defined as fever for more than 3 days with clinical deterioration necessitating a change in initial empiric antibiotic therapy, the occurrence of a severe side effect, or death occurring after at least 48 h of antibiotic treatment^26^. The rate of treatment failure was higher in subjects with aspiration risk than that in non-aspiration pneumonia (48.6% vs 19.1%, p < 0.001), and was associated with aspiration pneumonia (OR, 2.97; 95% CI 1.29–6.86) with neoplasia (3.25; 1.11–9.56), neurological disease (2.34; 1.07–5.13), and elevated monocytes (0.40; 0.20–0.80).

### Publication bias

**Figure 5:**
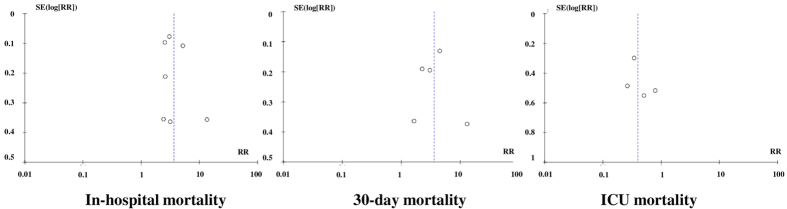
Funnel plots for in-hospital mortality, 30-day mortality and ICU mortality.

There appeared to be funnel plot asymmetry for in-hospital mortality (Fig. 5) suggesting the possibility of publication bias. Due to small number of studies included in each meta-analysis, Sterne’s test^31^ was not appropriate for detecting funnel plot asymmetry.

## Discussion

This systematic review suggests that aspiration risk is associated with greater in-hospital and 30-day mortality in subjects with CAP except, perhaps, in the ICU setting. Although there are insufficient data to perform a meta-analysis on long-term mortality, recurrent pneumonia, and hospital readmission, the few reported studies suggest that aspiration pneumonia is also associated with these outcomes.

Although aspiration pneumonia was significantly associated with in-hospital mortality when all studies were combined, multivariate analysis in individual studies suggested greater variability (Table 1). For example, Hayashi *et al*. reported that aspiration pneumonia did not remain as a significant risk factor for in-hospital mortality after multivariate analysis^63^, but that CURB-65 and ECOG PS increased in-hospital mortality in the same analysis. The CURB-65 includes confusion as a scoring item and the ECOG PS includes weakness as a severity of disability so these scoring systems may, in part, encompass aspiration risk factors. The Fujiki study reported the highest mortality of aspiration pneumonia at 59.1% with an especially strong association with in-hospital mortality (OR 49.9; 95% CI 6.23–398.94)^59^. Differences in study size, study population, and in the definition of what constitutes aspiration pneumonia may account for these markedly divergent results.

The risk ratio for 30-day mortality (3.57; 95% CI, 2.18–5.86) was similar to that for in-hospital mortality (4.67, 95% CI, 2.59–8.41) most likely because these outcomes are similar. Fernandez *et al*. focused on patients aged over 80 years and in these subjects, gram-negative pneumonia was the main risk factors for 30-day mortality^60^. HCAP patients, many of whom are elderly, may be at greater risk of death from aspiration pneumonia, but only one such study has been reported. In that study, aspiration pneumonia in subjects with HCAP was associated with an increased 30-day mortality compared to those with pneumonia but no aspiration (14.8% vs 4.3%, p = 0.025)^32^.

Paradoxically, in the studies that analyzed mortality in the ICU, aspiration risk was associated with a *lower* risk of death^67^,^72^,^73^,^74^. It was hypothesized that the younger study population, the use of intravascular volume repletion, the earlier appreciation and treatment of pneumonia in the ICU and close monitoring might improve prognosis^74^. It is also possible that patients with pneumonia who are admitted to the ICU are, on the whole, sicker and with comorbidities that might minimize any influence of aspiration on outcomes.

Aspiration risk was associated with all cause readmission, perhaps because many of these subjects were debilitated and had comorbidities^58^,^66^. In subjects with weakness or neurologic dysfunction (e.g. stroke), dysphagia and aspiration often continues even with careful medical management. In general, recurrent pneumonia within 3 to 5 years of an episode of CAP occurs in 9 to 12% of subjects with a median time to recurrence of 123 to 317 days and mortality ranging from 4 to 10%^76^. While some risk factors for aspiration pneumonia result from the natural course of aging^77^, a multidisciplinary approach might reduce the risk of aspiration pneumonia. El Solh *et al*. reviewed ways to prevent aspiration pneumonia and concluded that few data were available to guide an evidence-based approach to the prevention using drugs such as angiotensin-converting enzyme inhibitors or capsaicin^78^. The evidence relating to non-pharmacologic approaches; swallowing rehabilitation, thickening feeds, oral hygiene, gastroesophageal reflux management, and a head-up position are also limited, but the combination approach using pharmacologic and non-pharmacologic methods may be of value in high risk patients^79^,^80^.

To summarize our results, aspiration risk is associated with in-hospital and 30 day mortality outside the ICU, with long term mortality, all cause readmission, and recurrent pneumonia. Most of the studies demonstrated that aspiration pneumonia is an independent risk factor for these outcomes. There are several limitations to interpreting these data. All studies were observational and most are retrospective. This has the potential to introduce selection, measurement, and possibly publication bias. Due to the small number of publications for each outcome, we could not conduct Stem’s test or meta-regression analysis for confirm funnel plot asymmetry as a measure of publication bias, however, visual examination of the funnel plots suggested the possibility of bias for in-hospital mortality but not for other outcomes. Although we have provided specific *ad hoc* definitions for pneumonia and for aspiration risk; these are still somewhat subjective making case ascertainment challenging. If uniform criteria to establish aspiration risk can be developed and accepted for future studies, this will enable well controlled and appropriately powered studies to determine if interventions that can decrease aspiration risk, will also affect morbidity and mortality in this population.

## Additional Information

**How to cite this article**: Komiya, K. *et al*. Prognostic implications of aspiration pneumonia in patients with community acquired pneumonia: A systematic review with meta-analysis. *Sci. Rep.* **6**, 38097; doi: 10.1038/srep38097 (2016).

**Publisher's note:** Springer Nature remains neutral with regard to jurisdictional claims in published maps and institutional affiliations.

## Figures and Tables

**Table 1:** In-hospital mortality.

	Nationality	Design	Age, years	Prevalence of aspiration pneumonia	In-hospital mortality			Multivariate analysis	
					Aspiration pneumonia	Non-aspiration pneumonia	P value	OR/HR (95% CI)	P value
Riquelme 1996*	Spain	Retrospective	>65	9/101 (8.9%)	5/9 (55.6%)	21/92 (22.8%)	0.161	RR 7.93 (0.93–67.58)	0.058
Marrie 2003**	Canada	Retrospective	18–55	835/11684 (7.1%)	108/835 (12.9%)	268/10849 (2.5%)	<0.001	OR 4.4 (3.3–5.7)	<0.05
Fujiki 2007*	Japan	Prospective	>16	22/227 (9.7%)	13/22 (59.1%)	10/227 (4.4%)	<0.001	OR 49.9 (6.23–398.94)	<0.0001
Hsu 2011**	USA	Retrospective	adult	745/50758 (1.5%)	144/745 (19.3%)	3157/50013 (6.3%)	<0.001	NA	NA
Nakagawa 2013**	Japan	Retrospective	>65	484/960 (50.4%)	72/484 (14.9%)	28/476 (5.9%)	<0.001	ND	ND
Hayashi 2014**	Japan	Retrospective	>15	100/214 (46.7%)	25/100 (25.0%)	9/114 (7.9%)	0.001	HR 1.067 (0.509–2.238)	0.864
Lanspa 2015***	USA	Retrospective with propensity score	>18	451/5185 (8.7%)	104/451 (23.1%)	426/4734 (9.0%)	<0.001	OR 2.3 (1.56–3.45)	<0.001

HR; hazard ratio, NA; not assessed, ND; not described, OR; odds ratio, RR; relative ratio.

***good, **moderate or *poor quality assessed based on biases using the modified Hayden’s criteria.

**Table 2:** 30-day mortality.

	Nationality	Design	Age, years	Prevalence of aspiration pneumonia	30-day mortality			Multivariate analysis	
					Aspiration pneumonia	Non-aspiration pneumonia	P value	OR/HR (95% CI)	P value
Fernández 2003*	Spain	Prospective	>80	46/305 (15.1%)	7/30 (23.3%)	39/275 (14.2%)	0.183	ND	NS
Komiya 2013**	Japan	Retrospective	adult	116/637 (18.2%)	26/116 (22.4%)	9/521 (1.7%)	<0.002	HR 5.69 (2.3–14.4)	<0.001
Taylor 2013***	UK	Prospective	>18	186/1348 (13.8%)	32/186 (17.2%)	89/1162 (7.7%)	<0.001	OR 1.42 (0.87–2.33)	0.2
Lanspa 2013*	USA	Retrospective	>18	510/3094 (16.5%)	97/510 (19.0%)	109/2584 (4.2%)	<0.003	OR 3.46 (2.11–5.67)	<0.001
Jaoude 2014**	USA	Retrospective	adult	329/658 (50.0%)*	92/329 (28.0%)	30/329 (9.1%)	<0.004	ND	NS

HR; hazard ratio, ND; not described, NS; not significant, OR; odds ratio.

***good, **moderate or *poor quality assessed based on biases using the modified Hayden’s criteria.

**Table 3:** ICU mortality.

	Nationality	Design	Age, years	Prevalence of aspiration pneumonia	ICU mortality			Multivariate analysis	
					Aspiration pneumonia	Non-aspiration pneumonia	P value	OR/HR (95% CI)	P value
Leroy 1995**	France	Retrospective + Prospective	adult	47/299 (15.7%)	4/47 (8.5%)	81/252 (32.1%)	0.0007	ND	NS
Leroy 1996***	France	Retrospective + Prospective	>16	56/335 (16.7%)	4/56 (7.1%)	25/279 (9.0%)	0.7987	−0.37 (prognostic score by validation cohort)	<0.05
Leroy 1997**	France	Retrospective + Prospective	adult	116/505 (22.9%)	11/116 (9.5%)	108/389 (27.8%)	0.0002	NA	NA
Georges 1999**	France	Retrospective + Prospective	adult	20/505 (4.0%)	3/20 (15.0%)	34/114 (29.8%)	0.277	NA	NA

HR; hazard ratio, NA; not assessed, ND; not described, NS; not significant, OR; odds ratio.

***good, **moderate or *poor quality assessed based on biases using the modified Hayden’s criteria.

**Table 4:** All cause readmission and recurrent pneumonia.

	Nationality	Design and definition of readmission	Age, years	Prevalence of aspiration pneumonia	All cause readmission			Multivariate analysis	
					Aspiration pneumonia	Non-aspiration pneumonia	P value	OR/HR (95% CI)	P value
Taylor# 2013***	UK	Prospective Within 1y after discharge	>18	186/1348 (13.8%)	ND	ND	ND	HR 1.52 (1.21–1.91)	<0.05
Jaoude# 2014**	USA	Retrospective Within 30d after discharge	adult	329/658 (50.0%)	58/329 (17.6%)	22/329 (6.7%)	<0.001	OR 2.3 (1.3–14.7)	<0.05
	**Nationality**	**Design and definition of recurrent pneumonia**	**Age, years**	**Prevalence of aspiration pneumonia**	**Recurrent pneumonia**			**Multivariate analysis**	
					**Aspiration pneumonia**	**Non-aspiration pneumonia**	**P value**	**HR (95% CI)**	**P value**
Garcia 2009**	Spain	Prospective >2 episodes after discharge within 3y	adult	103/1556 (6.6%)	23/103 (22.3%)	201/1531 (13.1%)	0.0118	ND	NS
Taylor# 2013***	UK	Prospective >1 episode after discharge within 1y	>18	186/1348 (13.8%)	ND	ND	ND	HR 3.13 (2.05–4.78)	<0.05
Hayashi# 2014**	Japan	Retrospective >1 episode after treatment within 3 m	>15	100/214 (46.7%)	54/100 (54.0%)	18/114 (15.8%)	<0.001	HR 2.643 (1.523–4.586)	0.001

HR; hazard ratio, ND; not described, NS; not significant, OR; odds ratio.

***good, **moderate or *poor quality assessed based on biases using the modified Hayden’s criteria. ^#^Multiple outcome study.
